# Address of the Retiring President, Dr. W. H. Goddard

**Published:** 1868-02

**Authors:** 


### ADDRESS OF THE RETIRING PRESIDENT,

### DR. W. H. GODDARD.

Read before the Mississippi Valley Dental Association, at its annual meeting, March 4th, 1868.

GENTLEMEN OF THE MISSISSIPPI VALLEY DENTAL ASSOCIATION: I have not spent much time in the selection of a subject on which to address you. For I have preferred to submit myself directly and unreservedly to the auspices of this occasion, and to draw my inspiration from its sympathies and suggestions. Thus I have taken counsel of my heart rather than of my head; and I ask your attention to a few earnest words on "*The worth and dignity of the Dental profession, and the responsibility it imposes.*"

The Dental profession has three fundamental claims to high respect and attention. 1st. It demands scientific knowledge. 2d. It demands artistic skill. 3d. It demands a pure, true, worthy character. Let me invite your attention to a presentation of these points in some detail.

1st. The Dental profession demands scientific knowledge in those who undertake it. It has to do with a portion of the human body, that noblest of all the material works of God on earth. It is in close alliance with the provinces of both the surgeon and the physician, who have always, when worthy of their vocation, enjoyed an honorable station among the fraternity of scholars and scientific experts, and also in the foremost ranks of society at large.

The condition of the teeth is immediately affected, yes, controlled by the condition of the body; and I defy a man to undertake their repair in a proper manner, and advise in regard to their due care and regulation, without a thorough knowledge of the structure of the body, and those great laws of digestion, nutrition, circulation, nervous force and action, which regulate the economy of life, health and growth. Their own particular structure too, their laws of formation and growth, and the occasions and processes of their decay, constitute a beautiful and wonderful as well as necessary study; embracing principles of science as profound and comprehensive as those that pertain to any other portion of the human frame.

Here then do we establish for our profession a basis of true honor; a claim to public deference and respect, founded on the loftiest principles on which any class of men assert their right to scientific distinction, and to social position and regard. And what is there, I ask, to prevent us, as a body, from achieving the same rank and being rewarded with the same honors as others? What is there to interfere between us and the recognition of our deserts? What except our own falseness to our true position and claims? I appeal to you then, as men who prize the honor of your profession, to regard with jealous interest its relations to science on the one hand and to society on the other. I appeal to you to consecrate yourselves to the work of vindicating its noble and ennobling claims. And in order to do this, as the first step towards its purification and elevation, I appeal to you to be personally faithful to its high demands; I appeal to you as the second step, to discountenance, and seek earnestly to expel from their assumed position in our body, those empirics, whose ignorant charlatanry is continually bringing reproach and odium on our profession; who have taken it up and pursue it, not with any enthusiastic appreciation of it as a science and an art, but only with a sordid idea that it offers a cheap and ready way to secure a livelihood and to make money; who therefore have no science about them; no lofty principles of fealty to scientific truth in their practice; but who go on without heart or conscience in a course of ignorant pretension, blind to the mischief that they are likely to cause.

It will be worse than useless to publicly denounce these charlatans, and brand them with the epithets that will justly characterize them. For instantly the cry will be raised that our opposition is inspired by our selfish and sordid jealousy; that we merely fear the competition of these persons, and traduce and defame them, so as to crush them out of our way. Thus we should be instrumental only in securing for them sympathy first, and through that sympathy business; and should increase the very evil we were interested to abate.

But this much we can do; and it is a course that while it preserves our own dignity, will be gradually and decisively effective against the unworthy, and force them from the position which they disgrace; and that is, to be in all points faithful, true and high-minded ourselves.

Then when by our own scientific culture and ability, we shall have demonstrated the real foundations on which our profession rests, the contrasts we shall exhibit to the ignorance of these pretenders will not fail to impress itself on the public mind, and induce a withdrawal of patronage from them. May that time speedily come! Intensely conscious of the exalted relations of Dentistry to the truths and requisites of science, I have no tolerance for the charlatanry that so widely prevails under that name, that ought to cover nothing that is not enlightened and true. And I rejoice in the opportunity this occasion affords, to utter my voice in indignant tones on the subject, happy if through these fleeting words I can stimulate afresh the right motives that inspire you, and combine us in that faithful activity, which is essential to the purification and honor of our profession.

I am to speak in the second place, of our profession, as demanding artistic skill, and in this connection, as well you know, I can utter myself with the same positiveness as in connection with its relations to science.

The true, real Dentist is, of necessity, an exquisite artist. His work, to be neatly and effectively performed, requires instruments of the most cunning workmanship, handled with the utmost dexterity, in conformity to methods that involve the most finished processes of art. There is not a stronger contrast between the skill of the most finished artificer in any branch of delicate handicraft, and the botch-work of an unpracticed blunderer in the same, than between the admirable performances of an accomplished Dentist and the sham work of a rude unskilled pretender; and therefore, as in connection with our former consideration in respect to science, I make bold to speak with the utmost truth and freedom, and to urge the same demands upon you. As I pleaded with you then to respect and elevate your profession because of its scientific claims, so now I plead with you to respect and elevate it, because of its admirable alliances to art, and just in proportion to our adequate appreciation of it in this respect, must be our indignant recoil from the miserable unskillfulness of those who have adopted without either science to guide them, ~~or skill~~ properly to accomplish what they undertake.

It is the glory of a faithful artist, in whatever walk of art to make any sacrifice rather than do violence to those eternal principles that underlie all art. So it is the glory of a true Dentist to act for the real interest of his patient, although pecuniary loss may be the result. But these impostors whom I am denouncing, will prosecute a case that may be offered to them, for filthy lucre's sake, even although they know that it will be to the injury of the patient. What a wrong is done to the community every year, enormous in the aggregate, by bungling unskilled operators on teeth! How many an unsuspecting person, trusting to their lying advertisements, is deceived into trusting to their work, and supposing that all is right, the work properly and faithfully done, and his teeth secured from further decay and rendered serviceable; and yet, in a few months, perhaps in a few weeks or days even, the fraud on their confidence and their purses becomes manifest, the work proves worthless, and with injuries perhaps, that will interfere with their health and prove the ruin of their teeth, they experience the indignant conviction that they have been cheated and wronged, and learn to mistrust the honesty, skill, and high-mindedness of the whole fraternity.

Now what is to remedy this constant outrage on our profession, this fraud on the public? How are the public to be induced to discriminate between the two classes of practitioners, and employ and uphold only those who are accomplished and worthy?

The same difficulties beset the method of publicly denouncing these knavish bunglers, as in denouncing those who are void of the necessary knowledge. I can only repeat the recommendation already given, to crush them out by the superior weight of our pretensions and character. This much is certain, that no sane person who has been once abused and fleeced by these impostors will submit to undergo the cheat a second time; and out of these we shall soon have quite an army at our backs, to aid in the right guidance of public opinion. And beyond that, we must be content to work out the problem slowly and surely by our personal fidelity and skill.

Let every true Dentist place the word "Excelsior" on his banner as the only motto by which he will consent to be inspired and guided; let him pursue with devotion and conscientious fidelity his experimental career, wedding skill to science, thus on the one hand satisfying his conscience, and achieving excellent practical results; and thus let him draw in bold and unmistakable characters, the line between himself and the charlatan who is gulling an unsuspecting public. Honor will surely redound to whom honor is due!

And what is of equal importance, let us not confine our interests and attention to the present, but think of the future. We have our Dental Colleges. Let us foster them with our most ardent and persistent regards. Let us place their standard of requirement so high, and maintain it so rigidly in its lofty elevation, that their credentials in a young man's possession shall be his title deed to confidence, trust and respectability.

Let them send forth every year, classes so thoroughly furnished, both as to scientific knowledge and experimental artistic practice, that the profession shall be hedged about with the safeguards of honor; a noble corps of practitioners to sustain its integrity, and vindicate its claims; and its legitimate sons, protecting as well as serving the public, expel from society and position the bastards who dare to practice their rascality under its name!

I have used the word respectability, and this brings me to my third and last topic, viz.: The character that should belong to every Dentist who is worthy of his profession.

Had I the power of using language so as to express all that I feel on this point, I should be eloquent indeed. In default of that, let a few but earnest words interpret the intense feelings of my heart.

In what has gone before, the course of my argument has been continually drawing a distinction between honesty and dishonesty, candor and deception, real science and skill, and low false pretension, severally in the two classes which are nominally included in the ranks of our profession.

So far then, if no farther, there is a demand for high-mindedness, truth and honor in our fraternity.

So far if no farther, there is no place among us for the low selfish time-server and the designing knave. But I wish emphatically, to go farther,—I have in view the intimate relations and contacts into which our profession necessarily brings us and our patients. I have in view also, the influence that personal character always has and ought to have over the respectability and social standing of men. Never may the time come in America when character shall be divorced from reputation, and a man's place in society be irrespective of his moral deserts. And when I add to this general consideration the special ones before adverted to, that demand for our profession men of real sterling worth. I do entreat you, gentlemen, give to this vital point the attention it deserves.

If it be a disgrace to our profession to have in its ranks, ignorant charlatans and unskilled bunglers, it is still more disgraceful to have those whose low standard of morals infringes the requisites of Christian duty and social decorum.

Professional pride makes me denounce the former; but I can not but regard the fact of the latter, as no less than a *personal insult.* Every one of us indeed who is characterized by noble and pure aspirations, and is striving by an unsullied name and a title to the respect of the community, to honor his walk in life, must needs feel it as a reflection on himself, not only as a Dentist but as a man, when some base fellow, worthy only of social outlawry, usurps the name and strives to carry off the honors and emoluments of our profession.

Then let us set our standard high, and maintain it there. Let us make character a requisite for obtaining a degree in our Dental Colleges, as well as science and skill. Let us see to it as far as we can, that our patients, when they place themselves under our hands, are coming in contact with Christian gentlemen. Let us measure our own aspirations, and purposes by the demands and possibilities of our profession; and by our appreciation of its claims to unlimited respect and honor, make it sure that never in our persons or by our faithlessness, it shall receive the slightest wound!

Gentlemen, I have thus frankly expressed to you the feelings of my heart, as they have been called into exercise by this occasion. In a very unstudied manner I have endeavored to vindicate the claims of our profession.

I trust that your own hearts have gone along with me and that you have said, Amen—to my words. I trust that I have been giving voice to your own ardent and aspiring sympathies and desires, while I have been uttering my own.

And commending you and our profession to Him, to whom alone every man standeth or falleth, and appealing to every man who hears me to regard the credit of his profession and his own honor and character as infinitely more to be preferred than sordid acquisition.

I bid you all God speed in your several careers, wishing you health, happiness and true success.

